# In silico prediction of the animal susceptibility and virtual screening of natural compounds against SARS-CoV-2: Molecular dynamics simulation based analysis

**DOI:** 10.3389/fgene.2022.906955

**Published:** 2022-08-30

**Authors:** Priyanka Garg, Venkata Krishna Vanamamalai, Itishree Jali, Shailesh Sharma

**Affiliations:** National Institute of Animal Biotechnology (NIAB), Hyderabad, India

**Keywords:** SARS-CoV-2, natural compound screening, toxicity analysis, molecular dynamics simulation, RMSD, MMPBSA

## Abstract

COVID-19 is an infectious disease caused by the SARS-CoV-2 virus. It has six open reading frames (orf1ab, orf3a, orf6, orf7a, orf8, and orf10), a spike protein, a membrane protein, an envelope small membrane protein, and a nucleocapsid protein, out of which, orf1ab is the largest ORF coding different important non-structural proteins. In this study, an effort was made to evaluate the susceptibility of different animals against SARS-CoV-2 by analyzing the interactions of Spike and ACE2 proteins of the animals and propose a list of potential natural compounds binding to orf1ab of SARS-CoV-2. Here, we analyzed structural interactions between spike proteins of SARS-CoV-2 and the ACE2 receptor of 16 different hosts. A simulation for 50 ns was performed on these complexes. Based on post-simulation analysis, *Chelonia mydas* was found to have a more stable complex, while *Bubalus bubalis*, *Aquila chrysaetos chrysaetos*, *Crocodylus porosus*, *and Loxodonta africana* were found to have the least stable complexes with more fluctuations than all other organisms. Apart from that, we performed domain assignment of orf1ab of SARS-CoV-2 and identified 14 distinct domains. Out of these, Domain 3 (DNA/RNA polymerases) was selected as a target, as it showed no similarities with host proteomes and was validated in silico. Then, the top 10 molecules were selected from the virtual screening of ∼1.8 lakh molecules from the ZINC database, based on binding energy, and validated for ADME and toxicological properties. Three molecules were selected and analyzed further. The structural analysis showed that these molecules were residing within the pocket of the receptor. Finally, a simulation for 200 ns was performed on complexes with three selected molecules. Based on post-simulation analysis (RMSD, RMSF, Rg, SASA, and energies), the molecule ZINC000103666966 was found as the most suitable inhibitory compound against Domain 3. As this is an in silico prediction, further experimental studies could unravel the potential of the proposed molecule against SARS-CoV-2.

## 1 Introduction

Coronaviruses (CoVs) are responsible for various respiratory and intestinal infections. They affect a diverse group of livestock hosts and cause a wide number of diseases like acute and chronic hepatitis, gastroenteritis, progressive peritonitis, nephritis, etc. In humans, CoVs cause respiratory tract complications with varying degrees of severity (Berry et al., 2015). CoVs are single-stranded, positive-sense, enveloped RNA viruses. These viruses are not only widely spread in bats but are also reported in many other species, including cats, pigs, horses, birds, and humans (Zaki et al., 2012). They have been considered highly pathogenic to humans since the outbreak of severe acute respiratory syndrome (SARS) in 2002 and 2003 in China (Cui et al., 2019). After ten years, another virus—MERS-CoV—has emerged in the Middle Eastern countries (Berry et al., 2015). Both these viruses were directly transmitted from market civets and dromedary camels and were thought to have originated in bats. Many genetically diverse CoVs related to these viruses were also discovered in bats across the world (Cui et al., 2019). Most of these viruses were genetically similar to known human CoVs but were never reported in humans (Letko et al., 2020). Coronaviruses are classified into four genera: Beta-CoV, Alpha-CoV, Gamma-CoV, and Delta-CoV (Li, 2016). The β-CoVs are divided into four lineages–A, B, C, and D, where SARS-CoV and the newly emerging SARS-CoV-2 are included in lineage B, and lineage C includes MERS-CoV. All CoVs encode a surface glycoprotein called Spike protein, which binds to the host–cell receptor and mediates viral entry (Li, 2016; Letko et al., 2020). The spike proteins of SARS-CoV and SARS-CoV-2 share a high degree of homology and about 76.5% identity in amino acid sequences (Zhang et al., 2020a). Several poly proteins are expressed by SARS-CoV-2, including 15 non-structural proteins and four structural proteins [spike (S) protein, envelope (E) protein, membrane (M) protein, and nucleocapsid (N) protein] (Wu et al., 2020). Orf1ab is the longest in size and encodes the non-structural proteins which are essential for viral pathogenicity and have roles distinct from or in addition to those directly related to viral replication (Graham et al., 2008), while ORF2-10 encodes structural proteins and other auxiliary proteins. The S, M, and E proteins regulate the viral coat formation, while the N protein regulates the RNA genome packaging. Apart from that, S protein also promotes the host attachment and viral cell membrane fusion during the infection, thus determining the host range to some extent (Wu et al., 2020).

ACE2, a key enzyme in RAAS activation functions, acts as a receptor for SARS-CoV-2 (Vaduganathan et al., 2020). The binding of the spike protein to the host ACE2 receptors and its priming by TMPRSS2 protease plays a vital role in cell entry of the virus (Hoffmann et al., 2020). ACE2 is the surface protein of cells in various organs like the heart, lungs, kidney, arteries, and intestines. Thus, these are all susceptible to being infected by SARS-CoV-2 (Fu et al., 2020). However, the organ at the highest risk of damage is the lung due to abundance of ACE2 in lung alveolar epithelial cells (Behl et al., 2020).

Apart from humans, Beta-CoVs include many viruses that infect bats and domestic and wild animals. The SARS-CoV-2 outbreak was linked to the Wuhan animal market, which includes many species like seafood, birds, snakes, marmots, and bats. Some studies report that pangolins (*Manis javanica*) could be a potential intermediary host for SARS-CoV-2, with putative recombination signals found between pangolin, bat, and human coronavirus sequences (Jaimes et al., 2020). Thus, there is a need to study different animals for their susceptibility against SARS-CoV-2.

Along with this, many drugs are effective in *in vitro* activity against different CoVs, and no clinical evidence currently supports their usage in humans (Kalil, 2020). Previous research reports that natural compounds possess multiple biological activities, including antiviral properties (Xian et al., 2020). Thus, we wanted to identify a natural compound that binds and inhibits the proteins of SARS-CoV-2 and thereby prevents the virus. There are several *in silico* studies which propose potential inhibitors from diverse sources against distinct proteins of SARS-CoV-2 by using docking and simulations (Bhardwaj et al., 2021; Sharma et al., 2021; Singh et al., 2022). In this study, we screened a set of ∼1.8 lakh natural compounds to target Orf1ab of SARS-CoV-2. Furthermore, these selected natural molecules were compared on different computational parameters like docking, ADMET analysis, MD simulations (RMSD, RMSF, radius of gyration, SASA, and MM-PBSA binding energy), and a potential molecule was selected. The objective of the current research was to identify powerful inhibitor compounds that might effectively bind to Orf1ab of SARS-CoV-2. As this is an *in silico* study, further experimental studies could unravel the potential of the proposed molecule against SARS-CoV-2.

## 2 Material and methods

### 2.1 Predicting the animal susceptibility

#### 2.1.1 Protein structure modeling and validation

The sequences of ACE2 proteins of 16 different hosts (six livestock, nine wild animals, and humans) were downloaded from the NCBI. These animals were from the mammalian, reptilian, and avian classes. This study focused on the orders from the mammalian class, including Artiodactyla, Perrisodactyla, Chiroptera, Rodentia, Carnivora, Primates, Pholidota, and Proboscidea; orders from the reptilian class, including Testudines and Crocodilia; and orders from the avian class, including Acciptriformes and Galliformes (Table 1). The structures of 16 ACE2 were modeled by homology modeling using SWISSMODEL, which was accessed through the ExPASy online web server (Waterhouse et al., 2018). In SWISSMODEL, homology modeling comprises four main steps: identification of structural templates, alignment of the target sequence and template structures, model-building, and model quality evaluation. The spike protein of SARS-CoV-2 was obtained from the Zhanglab COVID-19 database with ID QHD43416 (Zhang et al., 2020b). Furthermore, the 16 ACE2 models were validated using the SAVES 6.0 web server (SAVES, 2022). SAVES is a set of different validating tools like VERIFY 3D, ERRAT2, PROVE, and PROCHECK. In Verify 3D, the models are assigned “PASS” if more than 80% of the amino acids have scored > = 0.2 in the 3D/1D profile. In ERRAT2, models with score more than 95% are considered to have good resolution. In PROVE, based on the percentage of buried atoms, the models are assigned: error (> 5%), warning (1%–5%), or pass (< 1%). In PROCHECK, models with over 85% of the residues in the core regions of the Ramachandran plot are considered to be good models.

**TABLE 1:** Table showing the list of 16 different organisms with their taxonomy IDs and their orders.

Order	Organism (Common name)	Taxonomy ID
Artiodactyla	*Bos taurus* (exotic cattle)	taxid:9913
	*Bubalus bubalis* (buffalo)	taxid:89462
	*Capra hircus* (goat)	taxid:9925
	*Ovis aries* (sheep)	taxid:9940
	*Sus scrofa* (pig)	taxid:9823
Perissodactyla	*Equus asinus* (donkey)	taxid:9,793
Chiroptera	*Rhinolophus ferrumequinum* (greater horseshoe bat)	taxid:59479
Pholidota	*Manis javanica* (Sunda pangolin)	taxid:9974
Carnivora	*Panthera tigris altaica* (Siberian tiger)	taxid:74533
Rodentia	*Cricetulus griseus* (hamster)	taxid:10029
Primates	*Homo sapiens* (human)	taxid:9606
Proboscidea	*Loxodonta Africana* (African elephant)	taxid:9785
Galliformes	*Gallus gallus* (chicken)	taxid:9031
Accipitriformes	*Aquila chrysaetos chrysaetos* (golden eagle)	taxid:8,962
Crocodilia	*Crocodylus porosus* (salt water alligator)	taxid:8,502
Testudines	*Chelonia mydas* (green sea turtle)	taxid:8,469

#### 2.1.2 Docking

The GRAMM-X (Tovchigrechko and Vakser, 2006) webserver was used for docking the spike protein with the modeled structures of ACE2 of host organisms. UCSF Chimera (Pettersen et al., 2004) was used for visualization of complex structures.

#### 2.1.3 Molecular dynamics simulations of ACE2 and spike

GROMACS is an open-source suite which is primarily designed for dynamical simulations of biomolecules. We used the standalone version 2021.1 of GROMACS (Abraham et al., 2015) for the molecular dynamics simulations (MDS) of spike and ACE2 complexes. CHARMM27 (Vanommeslaeghe et al., 2010), an atom force field, was assigned to the complexes using the TIP3P water model. All hydrogens in the input coordinate file were discarded and reassigned according to the force field. Furthermore, the complexes were then placed in the center of a solvated, dodecahedron box with a 1-nm box edge. The system was solvated using the spc216 water model, and to neutralize the system, sodium counter ions were added. The energy of the system was minimized by using the steepest descent minimization algorithm, and this step was required to sort out any clashes in starting structures that may have been caused during the generation of the system. Along with that, the Particle Mesh Ewald (PME) method (Essmann et al., 1995) with a cutoff of 1.2 nm was used to calculate the long-range electrostatic interactions of the system. Both the equilibration steps (NVT and NPT) and the production molecular dynamics simulation step were performed using a leap-frog integrator algorithm for 50 ns. Coordinates, velocities, and energies were saved every 2 fs. The short-range electrostatic and van der Waals cutoffs of the system were 1.2 nm, and all other parameters were used at default. Then, the trajectory files (tpr and trr) were obtained and used further to calculate the RMSD (root mean square deviation) value and analyze the potential energy, kinetic energy, and total energy of the complex.

### 2.2 Identification of natural compounds against SARS-CoV-2

#### 2.2.1 Domain assignment and target selection

The proteome information of SARS-CoV-2 shows that there are six open reading frames (orf1ab, orf3a, orf6, orf7a, orf8, and orf10), a spike protein, a membrane protein, an envelope small membrane protein, and a nucleocapsid protein (Zhang et al., 2020c). In terms of size, orf1ab is the largest, having 7,096 amino acids. Since the outbreak of COVID-19, orf1ab and spike protein were the widely studied proteins of SARS-CoV-2 (Angeletti et al., 2020; Zolfaghari Emameh et al., 2020). We downloaded the orf1ab sequence from the NCBI (QIB84672.1) and performed domain assignment using the SUPERFAMILY database (Gough et al., 2001) to predict the structural and functional domains of orf1ab.

Then, the sequence analysis study was performed to identify the similarities between the proteomes of the 16 different host organisms and the obtained domains of orf1ab using BLASTp (Altschul et al., 1990) search at default parameters. Then, based on the similarities with the host proteome and annotation, the domains containing no similarities with all the 16 organisms were selected as target domains.

#### 2.2.2 Protein structure modeling and validation of the modeled structures

The 3D structures of the selected domain sequences were modeled using the trRosetta online web server (Yang et al., 2020). It builds the 3D structure based on direct energy minimization with a restrained Rosetta. The restraints are predicted by a deep neural network and include inter-residue distance and orientation distributions. Furthermore, these modeled structures were validated using SAVES 6.0 as mentioned above in Section 2.1.1 and the Prosa web server (Wiederstein and Sippl, 2007). The stereochemical accuracy of the modeled structure of selected target domains and their overall structural geometry was confirmed by using the Procheck online server (Laskowski et al., 1993). To assess the stability of the model and validate the residues, Ramachandran plot statistics were examined. The ProsaWeb online server was used to evaluate the model’s overall quality (Wiederstein and Sippl, 2007). We also performed Z-score analyses for the modeled structure of the selected target domains.

#### 2.2.3 Virtual screening of natural compounds

We downloaded publicly available ∼1.8 lakh NCs from the ZINC database (Sterling and Irwin, 2015) and virtually screened against modeled structures of selected domains by using Autodock4 (Morris et al., 2009) on default parameters. First, we prepared the coordinate file by including the atomic partial charges and atom types (PDBQT), followed by the Autogrid generation by embedding the protein in a three-dimensional grid, and a probe atom was placed at each grid point. Finally, AutoDock was run several times to provide 10 docked conformations for each ligand, and analysis of the Estimated Free Energy of Binding (kcal/mol), Inhibition Constant, Ki (nM) and the consistency of results was combined to identify the best pose. As there are a large number of molecules being screened, we have used the in-house python script for automation. Then, the top 10 NCs were selected based on the binding energy of the complex and were further studied.

#### 2.2.4 Ligand-based ADME prediction

The pharmacokinetic properties such as absorption, distribution, metabolism, and excretion of the selected 10 natural compounds were evaluated using Lipinski’s rule of five (Lipinski et al., 2001). According to this rule, a compound which satisfies at least four characteristics out of the five, such as molecular weight between 150 to 500 Daltons, hydrogen bond donors less than or equal to 5, hydrogen bond acceptors less than or equal to 10, lipophilicity less than 5, and molar refractivity between 40 and 130, can be considered to have optimal drug-like behavior. In this study, the SwissADME (Daina et al., 2017) web server was used to analyze the pharmacokinetic properties of the top 10 selected natural compounds. The molecules that satisfy Lipinski’s condition were considered the ideal drug candidates.

#### 2.2.5 Prediction of toxicological properties

Due to the fact that toxicity is a primary concern when administering any medication, the toxicological characteristics of the chosen natural compounds were also predicted. The admetSAR web server (Cheng et al., 2012) and pkCSM web server (Pires et al., 2015) were used for prediction of toxicological properties of the molecules. Ames toxicity, maximum tolerated dose (human), carcinogenicity, inhibitory effects on human ether-a-go-go-related gene (hERG), and Oral Rat Acute Toxicity (LD50) were predicted. The canonical smiles of the NCs were used as the input to the web servers.

#### 2.2.6 Protein–ligand interaction analysis

Internal structural characteristics of the ligand-binding pockets and cavities are essential for drug discovery because they involve drug molecule and protein interactions, which are critical for the protein function mechanism. Two different algorithms—D3Pockets (Chen et al., 2019) and Fpocket (Le Guilloux et al., 2009)—were applied to study the ligand binding pockets in depth. D3Pockets were used to detect and analyze the dynamic properties of ligand binding pockets on targeted proteins. Fpocket was used for detection of protein pockets (cavity) based on the Voronoi tessellation algorithm. The results of D3Pockets and Fpockets were visualized by using Pymol (Yuan et al., 2017) and UCSF Chimera (Pettersen et al., 2004). Apart from this, we plotted 2D interaction patterns, including H-bonds and hydrophobic interactions between the receptor and ligand, using LigPlot plus v2.2 (Laskowski and Swindells, 2011).

#### 2.2.7 Molecular dynamics simulations of natural compounds and selected domains

The molecular dynamics simulations (MDS) of the selected natural compounds and domains of orf1ab were performed for 200 ns by using GROMACS standalone version 2021.1. In CHARMM27 (Vanommeslaeghe et al., 2010), all atom force fields were assigned to receptor structures using the TIP3P water model. All hydrogens in the input coordinate file were discarded and reassigned according to the force field. The selected ligands of the selected target domains of orf1ab were prepared for MDS by using the SwissParam (Zoete et al., 2011) web server. The solvation, neutralization, and energy minimization steps were performed using the same parameters as mentioned above in Section 2.1.2. Then, both the equilibration steps (NVT and NPT) and the production molecular dynamics simulation step were performed using a leap-frog integrator algorithm for 200 ns. Finally, the trajectory files were used to calculate the RMSD, RMSF, radius of gyration, SASA, and H-bonds and analyze the potential energy, kinetic energy, and total energy of the complex.

#### 2.2.8 Binding free energy calculation (Molecular Mechanics Poisson-Boltzmann Surface Area)

The Molecular Mechanics Poisson-Boltzmann Surface Area (MM-PBSA) approach was utilized to evaluate the binding free energies of all the three natural compounds with Domain 3 of Orf1ab to determine complex stability (Kumari and Dalal, 2021). The gmx_MMPBSA (Valdés-Tresanco et al., 2021) is a tool based on AMBER’s MMPBSA.py which is used to perform binding free energy calculations using AMBERTOOLS (Case et al., 2022). We have calculated the binding free energy using the C2 entropy and a nonlinear PB model. As per the manual, when running a nonlinear PB solver, the polar solvation energy term EPB in the output file is set to zero, while the electrostatic energy term includes both the reaction field energy (EPB) and the Coulombic energy (EEL) (Valdés-Tresanco et al., 2021). Evaluation of binding free energy implicates enumeration of van der Waals, electrostatic + polar, and solvent-accessible surface area energies. The binding energy was computed by using the following equation:

$$\Delta G_{\text{binding}}{=G}_{\text{complex}}-\left(G_{\text{receptor}}{+G}_{\text{ligand}}\right).$$

The term “G_complex_” refers to the total binding energy of the complex, “G_receptor_” to the binding energy of the free receptor, and “G_ligand_” to the binding energy of the unbound ligand.

## 3 Results

### 3.1 Predicting the animal susceptibility

#### 3.1.1 Protein structure modeling and validation

The 3D structures of the ACE2 receptors of the 16 different hosts were modeled with the 6LZG ACE2 model from the PDB database as a template using SWISSMODEL (Waterhouse et al., 2018), which was accessed through the ExPASy web server. The modeled structures were saved as PDB files, and the information of sequence identity, sequence similarity, and coverage is mentioned in Supplementary Table S1. After homology modeling using SWISSMODEL, the 16 ACE2 models were validated using SAVES (SAVES, 2022). The homology modeled structures analyzed in this study revealed no “error” in PROVE. The majority of the homology modeled structures had scores of > 90% in PROCHECK and > 95% in ERRAT2, indicating that the models were adequate for future investigation. Verify 3D has assigned “PASS” to all the models (Supplementary Table S2).

#### 3.1.2 Docking

**FIGURE 1:**
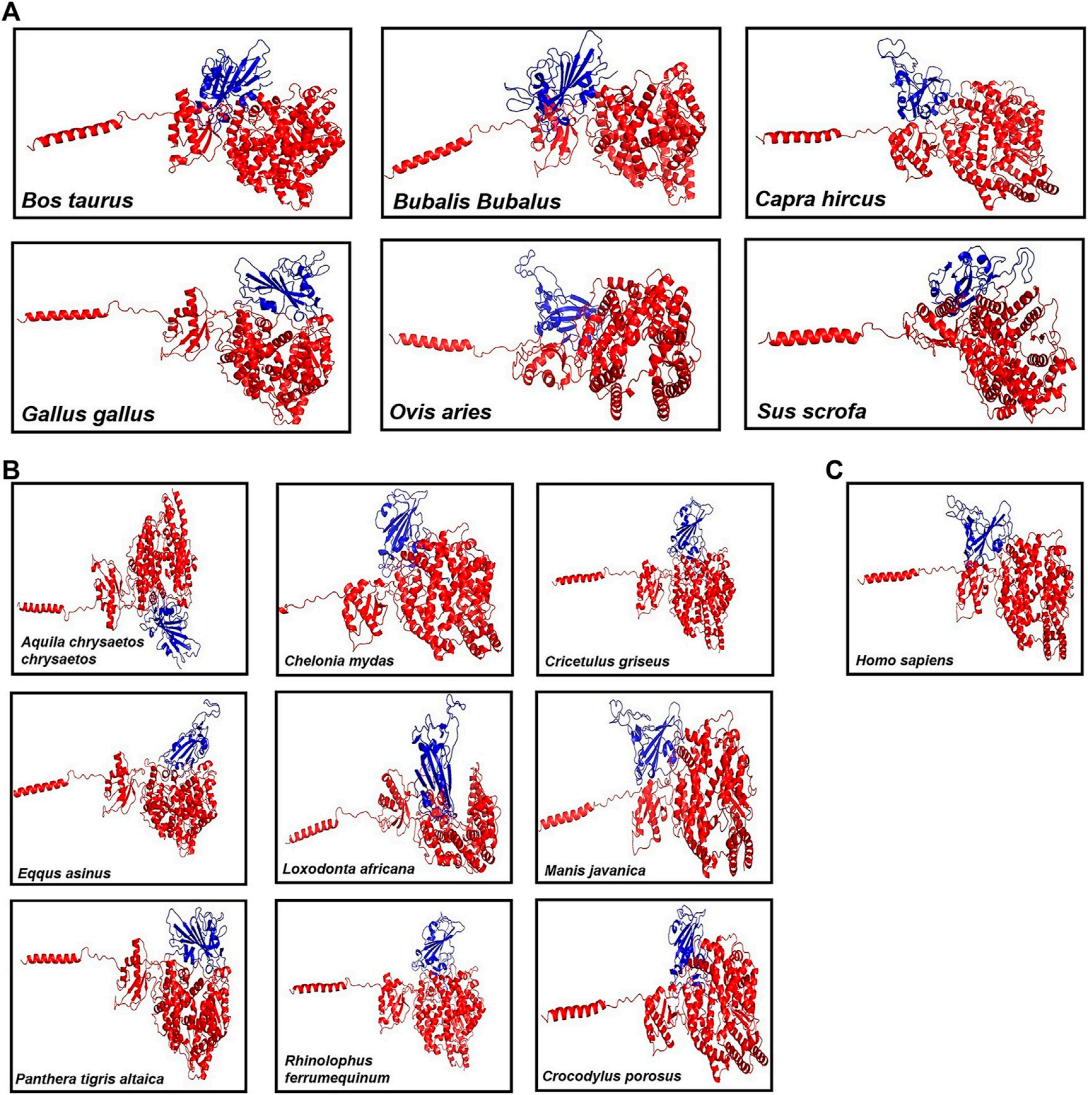
Figure showing the 3D models of the ACE2 receptor with spike protein of **(A)** six Livestock animals, **(B)** nine wild animals, and **(C)** human.

Then, these structures were docked with spike protein using GRAMM-X (Tovchigrechko and Vakser, 2006). The 16 ACE2–spike complexes were divided into three categories: first with six livestock animals, second with nine wild animals, and third with human complexes. The docked complexes of six livestock animals are shown in Figure 1A, of nine wild animals are shown in Figure 1B and those of humans are shown in Figure 1C.

#### 3.1.3 Molecular dynamics simulations of ACE2 and spike

**FIGURE 2:**
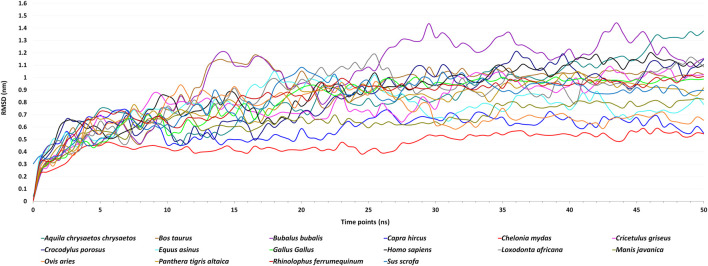
Figure showing Root Mean Square Deviation (RMSD) fluctuations of the spike–ACE2 receptor complexes of 16 host organisms for the MD simulations of 50 ns.

The molecular dynamics simulations of 16 ACE2 and spike complexes were performed separately using a GPU accelerated system for 50 ns. The energy minimization of these complexes was performed successfully, and it was observed that the complexes had no steric hindrance. The Epot was found to be negative and Fmax was found to be less than emtol (1,000.0) for all the complexes. After the two equilibration phases, the system was found to be well-equilibrated at 300 K temperature and 1 bar pressure. The trajectory files of the production MD step were used for further analysis. Based on the RMSD plot (Figure 2), *Chelonia mydas* was found to have a more stable ACE2-spike complex than other organisms in the study, in a range of 0.4 nm–0.6 nm, followed by *Capra hircus* in a range of 0.5 nm–0.7 nm and *Manis javanica* in a range of 0.6 nm–0.8 nm. In other organisms, after 25 ns, *Gallus* and *Rhinolophus ferrumequinum* were found to show stable RMSD between 0.9 nm and 1.0 nm. After 30 ns, *Ovis aries* showed RMSD between 0.6 nm and 0.8 nm and *Bos taurus* showed RMSD between 1.0 nm and 1.1 nm, while *Bubalus bubalis*, *Aquila chrysaetos chrysaetos, Crocodylus porosus*, and *Loxodonta africana* were found to be the least stable complexes with more fluctuations than all other organisms.

Different energies during the entire dynamics simulation were obtained, and it was found that the potential energy remained negative, kinetic energy remained positive, and total energy was observed to be negative throughout the simulation. This showed that *Chelonia mydas* was found to have a more stable ACE2-spike complex than all other organisms in the study. Thus, we assume *Chelonia mydas* to be more susceptible to SARS-CoV-2 infection, as reported in previous studies (Liu et al., 2020; Zhao et al., 2020).

### 3.2 Identification of natural compounds against SARS-CoV-2

#### 3.2.1 Domain assignment and target selection

By virtue of its size, orf1ab had the majority of the functional domains in the SARS-CoV-2 proteome. A total of 14 domains were obtained by using the SUPERFAMILY database. Details showing domains with boundaries, length, and annotation are mentioned in Table 2. Proteases, polymerases, NSPs, hydrolases, and methyltransferases were the major functional domains of orf1ab. These domains covered 31.17% (2,212 aa) of orf1ab, ranging from 13 to 6,793 amino acids.

**TABLE 2:** Table representing the 14 domains of orf1ab sequence (QIB84672.1) which is assigned by Superfamily database.

Domain number	Region	Length	Superfamily (annotation)
Domain Number 1	3,264–3,568	304	Trypsin-like serine proteases
Domain Number 2	4,889–5,090	201	DNA/RNA polymerases
Domain Number 3	5,119–5,282	163	DNA/RNA polymerases (RdRp)
Domain Number 4	6,453–6,641	188	S-adenosyl-L-methionine-dependent methyltransferases
Domain Number 5	3,980–4,133	153	Coronavirus NSP8-like
Domain Number 6	6,641–6,793	152	EndoU-like
Domain Number 7	4,149–4,250	101	Replicase NSP9
Domain Number 8	13–127	114	SARS Nsp1-like
Domain Number 9	4,259–4,381	122	Coronavirus NSP10-like
Domain Number 10	3,860–3,941	81	Coronavirus NSP7-like
Domain Number 11	819–929	110	NSP3A-like
Domain Number 12	1,031–1,187	156	Macro domain-like
Domain Number 13	5,600–5,914	314	P-loop containing nucleoside triphosphate hydrolases
Domain Number 14	1,567–1,620	53	NA

A BLASTp (Altschul et al., 1990) search of 14 domain sequences was launched at default parameters against the proteomes of 16 different hosts from mammalian, reptilian, and avian classes. Supplementary Table S3 shows the complete BLASTp results with annotations. Domains 1, 3, 5, and 10 had no similarity with all 16 species. On the other hand, Macro domain-like (domain 12) and P-loop-containing nucleoside triphosphate hydrolases (domain 13) had similar proteins in all 16 species. All the other domains have similarities with some of the host organisms. Supplementary Figure S1 shows the number of similarities between domains and proteomes of 16 hosts.

Out of the four domains (1, 3, 5, and 10) with no similarities, domain 3 with annotation DNA/RNA polymerases (RdRp) was chosen as the target as it plays a vital role in viral replication (Table 2). It should also be noted that the RdRp domain was used as a target for the widely known drug remdesivir (Lo et al., 2020; Nguyen et al., 2020). Additionally, previous experiments have shown that blocking viral RNA polymerases might considerably reduce viral loads in the lung tissue of mouse infected with MERS-CoV, increasing lung function and lessening the pathological harm to lung tissue (Rakib et al., 2021).

#### 3.2.2 Protein structure modeling and validation of the modeled structures

**FIGURE 3:**
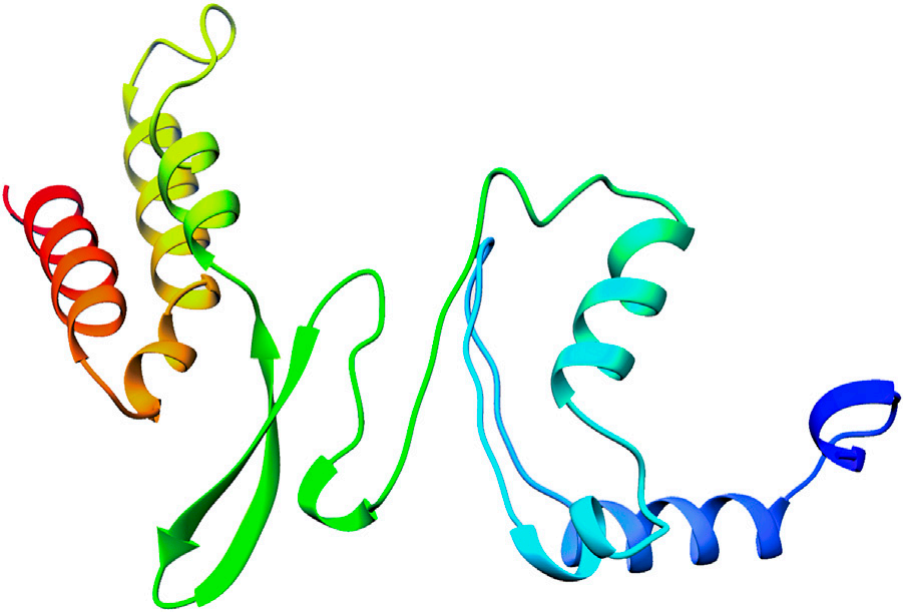
Figure showing the 3D structure of domain 3 of orf1ab predicted by the trRosetta online web server.

**FIGURE 4:**
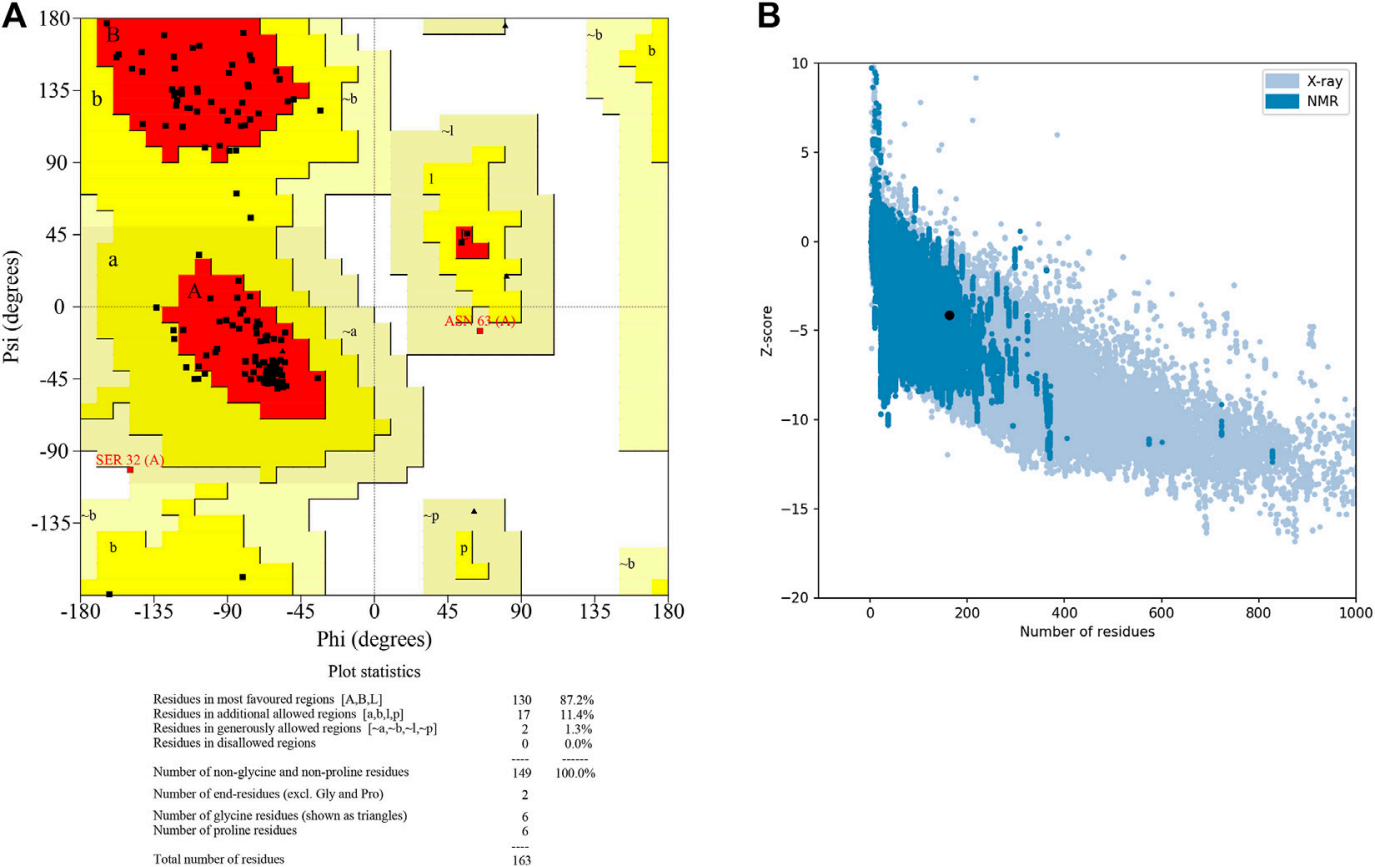
Figure showing the **(A)** Ramachandran plot and **(B)** Z score plot of domain 3 of orf1ab.

The 3D structure of selected domain 3 predicted by the trRosetta online web server (Yang et al., 2020) is shown in Figure 3. After homology modeling using trRosetta, the 3D model of the selected domain 3 was validated using SAVES. The homology modeled structure that was used in this study showed no “error” in PROVE. The homology modeled structure had a > 97% score in ERRAT2, showing the model was good enough for further analysis. The model was assigned “PASS” by Verify 3D (Supplementary Table S2). Along with that, the modeled structure of domain 3 was validated using PROCHECK by geometrical conformations and stereochemical quality assessments. The Ramachandran plot of the modeled structure of domain 3 indicated that 87.2% of residues were in the most favorable region, 11.4% were in the allowed region, 1.3% were in the generously allowed region, and 0.0% were in the disallowed region **(** Figure 4A **)**. In addition, the ProsaWeb online tool predicted the Z-score of the modeled structure of domain 3 was −4.14, which indicates that the model exemplifies the quality of a nuclear magnetic resonance (NMR) structure (Figure 4B).

#### 3.2.3 Virtual screening of natural compounds

Autodock4 (Morris et al., 2009) was used to virtually screen natural compounds against Domain 3 at default parameters with 10 conformations for each ligand. Out of them, we selected the best conformation based on the estimated free energy of binding and inhibition constant. Then, we selected 10 ligands based on the binding energy with the receptor using in-house python scripts and obtained structures of the receptor separately with the selected top 10 natural compounds in PDB format. The protein–ligand interaction details like Estimated Free Energy of Binding (kcal/mol), Estimated Inhibition Constant (nM), Final Intermolecular Energy (kcal/mol), Torsional Free Energy (kcal/mol), Residues with H-Bonds, and Torsional Degree of freedom of ten confirmations of the top 10 ligands are mentioned in Supplementary Table S4.

#### 3.2.4 Ligand-based ADME prediction

Out of top 10 NCs, only three molecules satisfied both Lipinski’s rule of five and Veber’s rule. The Absorption, Distribution, Metabolism, and Excretion (ADME) properties are shown in Table 3. The molecules ZINC000020413317, ZINC000103666966, and ZINC000030883119 fulfilled four out of the five characteristics, such as molecular weight between 150 to 500 Daltons, hydrogen bond donors less than or equal to 5, hydrogen bond acceptors less than or equal to 10, and lipophilicity less than 5 except molar refractivity, which was found to be more than 130.

**TABLE 3:** Table showing the ADME result by using SwissADME.

Molecule	Lipinski’s filter						Veber’s filter	
	Molecular weight (g/mol)^a^	Num. of H-bond acceptor^b^	Num. of H-bond donor^c^	MlogP^d^	Molar refractivity^e^	Lipinski’s rule of five violation^f^	Num. of rotatable bond^g^	TPSA (Å^2^)^h^
ZINC000085550032	504.79	3	3	5.66	159.57	3	3	44.29
ZINC000085550048	475.75	2	2	6.36	151.96	2	2	32.26
ZINC000020413317	468.63	5	2	2.98	139.41	1	6	74.94
ZINC000106920451	539.66	5	1	4.14	162.73	2	7	79.98
ZINC000008382440	540.73	4	0	5.49	159.55	3	7	60.44
ZINC000103666966	468.63	5	2	2.98	139.41	1	6	74.94
ZINC000253502470	574.71	9	5	1.33	152.83	2	9	165.75
ZINC000085489869	536.87	0	0	8.96	184.43	3	10	0
ZINC000030883119	422.61	5	0	4.04	135.5	1	6	45.4
ZINC000085550027	502.77	3	3	5.57	159.09	3	3	44.29

a Molecular weight less than 500 Dalton.

b Less than or equal 10 hydrogen bond acceptors.

c Less than or equal 5 hydrogen bond donors.

d High lipophilicity (expressed as LogP) less than 5.

e Molar refractivity should be between 40 and 130.

f Lipinski’s rule of five violations less than or equal 1.

g Less than or equal 10 rotatable bonds.

h Topological polar surface area (TPSA) less than or equal 140 Å2.

#### 3.2.5 Prediction of toxicological properties

The toxicological properties of the ligand need to be assessed to ensure the efficacy level and safety of the top 10 natural molecules. The toxicological characteristics of the top 10 natural compounds of Domain 3 were predicted using the online web servers – admetSAR and pkCSM (Table 4). The results showed that all the top 10 natural compounds were likely non-carcinogenic. Moreover, the Ames toxicity test was positive for only one molecule, i.e., ZINC000085489869, proving that the majority of the substances did not present a mutagenic concern. The acute oral toxicity values for all the compounds fall into Class III, which includes compounds with LD_50_ values greater than 500 mg/kg but less than 5000 mg/kg and were typically thought to be suitable for use as drugs (Chander et al., 2016). All the compounds showed marginal rat acute toxicity, with a median lethal dose (LD_50_) ranging from 1.976 to 3.267 mol/kg. Apart from this, the maximum recommended tolerated dose (MRTD) that estimates the threshold of dose producing an “acceptable level of toxicityˮ was found to be higher than the cut-off (0.477 log mg/kg/day) for ZINC000008382440, i.e., 0.998 and lower than the cut-off for the other nine compounds. Thus, out of the 10 examined natural compounds, except two molecules, i.e., ZINC000085489869 and ZINC000008382440, all others satisfied the selected toxicological parameters. Finally, based on the ADME and toxicity parameters, three molecules, i.e., ZINC000020413317, ZINC000103666966, and ZINC000030883119 were selected and analyzed further.

**TABLE 4:** Table showing the toxicological properties of top 10 ncs by using admetSAR and pkCSM web servers.

Molecule	AMES toxicity	Carcinogen	Acute oral toxicity	Oral Rat acute toxicity (LD50) mol/kg	Max. tolerated dose (human) mg/kg/day
ZINC000085550032	Non-Ames toxic	Non-carcinogens	III	3.267	−0.156
ZINC000085550048	Non-Ames toxic	Non-carcinogens	III	2.934	−0.234
ZINC000020413317	Non-Ames toxic	Non-carcinogens	III	2.656	−1.025
ZINC000106920451	Non-Ames toxic	Non-carcinogens	III	2.797	0.308
ZINC000008382440	Non-Ames toxic	Non-carcinogens	III	2.627	0.998
ZINC000103666966	Non-Ames toxic	Non-carcinogens	III	2.656	−1.025
ZINC000253502470	Non-Ames toxic	Non-carcinogens	III	3.039	−0.485
ZINC000085489869	Ames toxic	Non-carcinogens	III	1.976	−0.071
ZINC000030883119	Non-Ames toxic	Non-carcinogens	III	3.126	−0.113
ZINC000085550027	Non-Ames toxic	Non-carcinogens	III	3.261	−0.229

#### 3.2.6 Protein–ligand interaction analysis

**FIGURE 5:**
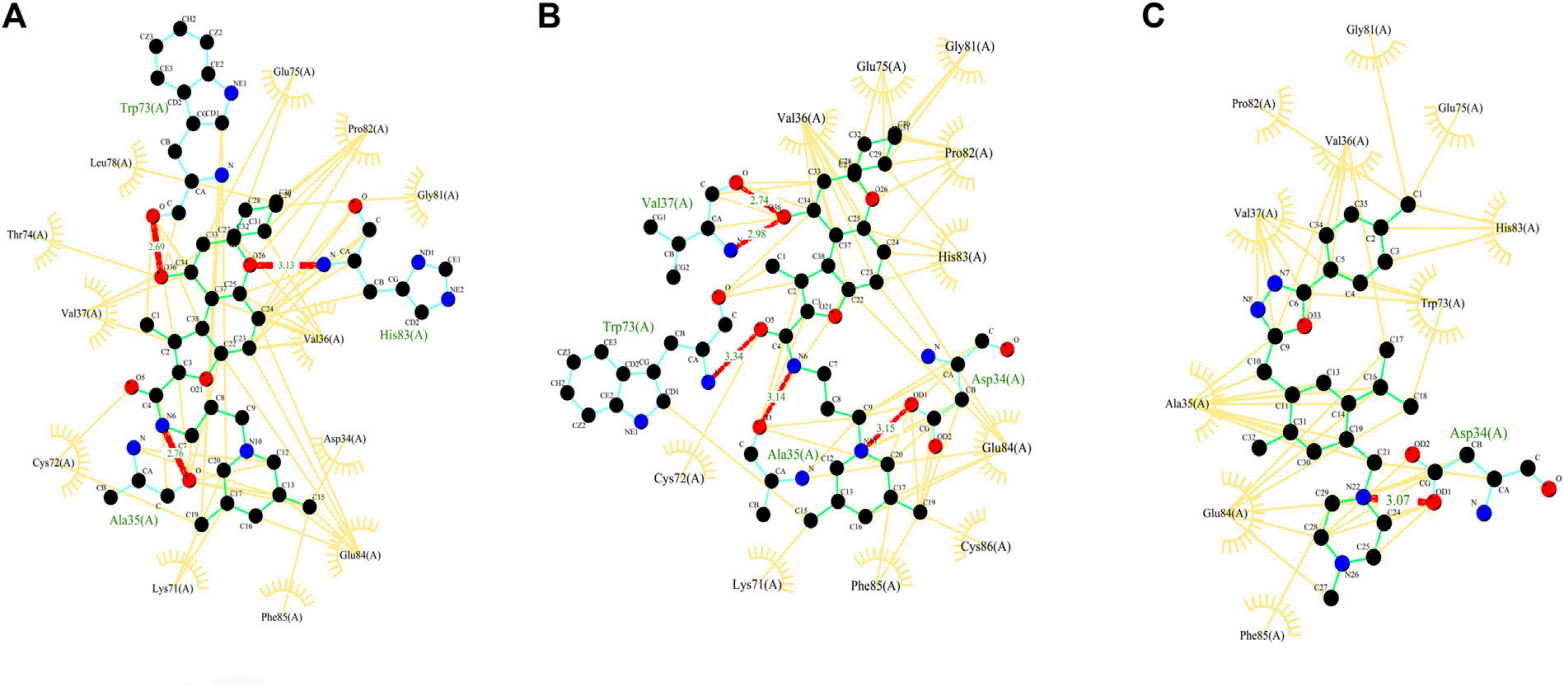
Figure showing the 2D interaction patterns (hydrogen bonds and hydrophobic interactions) of three selected complexes–molecules **(A)** ZINC000103666966, **(B)** ZINC000020413317 and **(C)** ZINC000030883119.

With D3Pockets and Fpocket webservers, pockets were predicted for the complex of domain 3 and selected three NCs. It was observed that all the three molecules were harbored inside the pocket of the receptor, which is shown in Supplementary Figure S2. Apart from this, we analyzed the interaction patterns of the selected molecules with the receptor using the LigPlot + tool. In these molecules (ZINC000103666966, ZINC000020413317, and ZINC000030883119), Asp 34, Ala 35, Trp 73, and His 83 were involved in protein–ligand binding. These are same as reported previously (Aftab et al., 2020), and the amino acids Asp761, Ala762, Trp800, and His810 were involved in ligand binding. The positions of amino acids were different as we considered only the domain part of RdRp as annotated by the Superfamily database. The complex of receptor with ZINC000103666966 showed H-bonds with three amino acids (Ala35, Trp73, and His83) and hydrophobic interactions with 12 amino acids (Asp34, Val36, Val37, Lys71, Cys72, Thr74, Glu75, Leu78, Gly81, Pro82, Glu84, and Phe85), while the complex with ZINC000020413317 showed H-bonds with four amino acids (Asp34, Ala35, Val37 and Trp73) and hydrophobic interactions with 10 amino acids (Val36, Lys71, Cys72, Glu75, Gly81, Pro82, His83, Glu84, Phe85, and Cys86). The complex with ZINC000030883119 showed an H-bond with one amino acid (Asp34) and hydrophobic interactions with 10 amino acids (Ala35, Val36, Val37, Trp73, Glu75, Gly81, Pro82, His83, Glu84, and Phe85) as mentioned in Figure 5.

#### 3.2.7 Molecular dynamics simulations of natural compounds and selected domains

The Molecular Dynamics Simulations (MDS) of domain 3 of orf1ab and selected NCs were performed using the Standalone version of GROMACS (Abraham et al., 2015) following standard parameters of equilibration and energy minimization for 200 ns. The energy minimization of these complexes was performed successfully for 1,078, 1,030, and 702 steps for the complexes with ZINC000103666966, ZINC000020413317, and ZINC000030883119, respectively, and potential energy was found to be −752995.625, −752292.3125 and −719172.125, respectively. It was observed that the complexes had no steric hindrance and F_max_ was found to be less than 1,000.0 for all the complexes. After the equilibration phase -I (nvt), the temperature of the system was found to be well-equilibrated at approximately 300 K, and then the equilibration phase -II (npt) pressure of the system was found to be equilibrated at approximately 1 bar. The trajectory files of the production MD step were used for further analysis.

##### 3.2.7.1 RMSD

**FIGURE 6:**
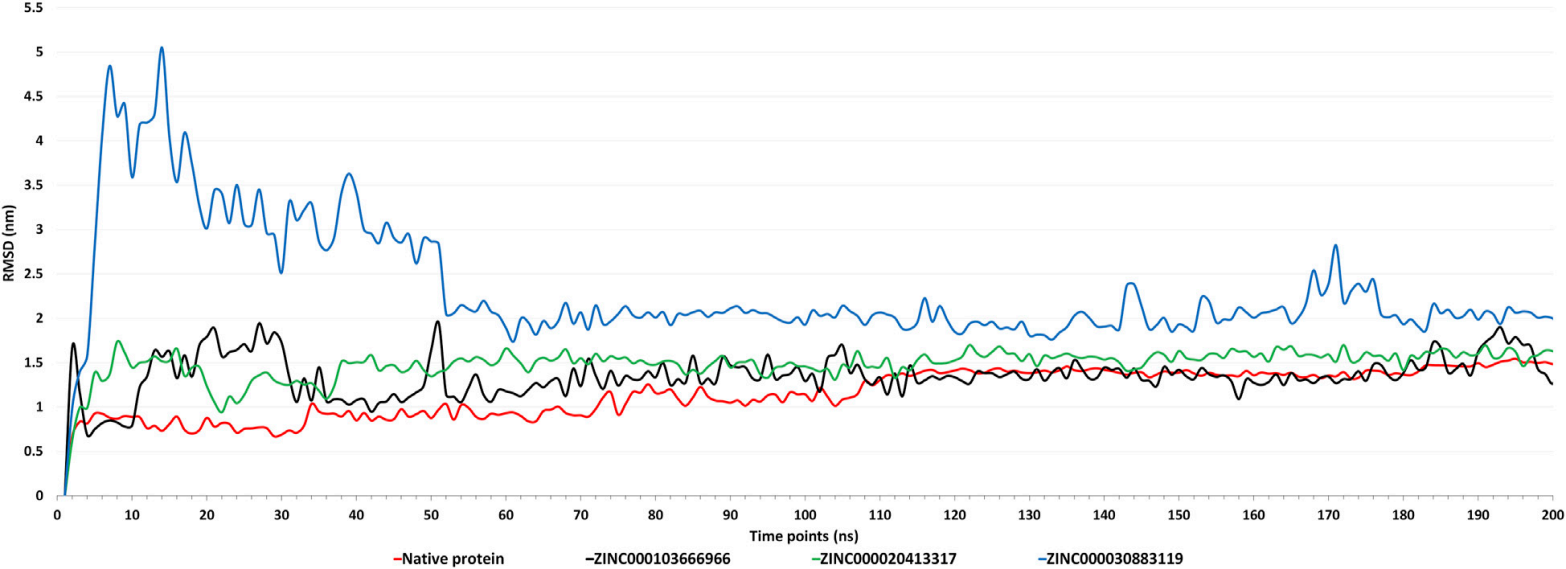
Figure showing the graphs of Root Mean Square Deviation (RMSD) of native protein (apoform) and complexes with molecules ZINC000103666966, ZINC000020413317, and ZINC000030883119 for the MD simulations of 200 ns.

By monitoring the RMSD during the molecular simulation, the dynamics variation in Cα backbone of the complexes was detected (Kumari and Dalal, 2021). Based on the RMSD plot (Figure 6), all the three complexes attained stability after 50 ns till 200 ns. The RMSD of native protein was in the range of 0.8–1.5 nm, with an average RMSD of 1.266 nm and a maximum fluctuation of up to 1.544 nm. After 100 ns, there were very few fluctuations in RMSD. The RMSD of the complex with ZINC000103666966 was in the range of 1–1.9 nm, with an average RMSD of 1.37 nm and a maximum of 1.95 nm. While in the case of the ZINC000020413317 complex, RMSD was found in the range1.3–1.7 nm, with an average RMSD of 1.538 nm and a maximum of 1.737 nm. The complex with ZINC000030883119 showed an initial spike of RMSD up to 5.05 nm, but after 50 ns, RMSD was found in the range of 1.73–2.83 nm, with an average of 2.04 nm and maximum up to 5.049 nm. Overall, RMSD results suggest that the complex with ZINC000103666966 had RMSD close to the native protein. Thus, the complex of domain 3 with molecule ZINC000103666966 was found to be comparatively more stable than the complex with molecules ZINC000020413317 and ZINC000030883119.

##### 3.2.7.2 RMSF

RMSF analysis shows the flexibility and fluctuations of the protein in complex compared to the native protein. The higher RMSF values indicate that there are loosely organized loops and turns, while lower flexibility shows the secondary structures like helixes and sheets (Kumari and Dalal, 2021). The RMSF plot (Supplementary Figure S3) showed that most of the residues in native protein had higher fluctuations than those in the complexes, showing that binding of ligands had decreased the fluctuations in the protein, were well-fitted, and formed a stable complex. Apart from that, the residues involved in the binding were also found to be less fluctuant in the complex than the native protein.

##### 3.2.7.3 Radius of gyration

The radius of gyration (Rg) analysis shows the overall compactness of the protein during the simulation. Rg demonstrates the moment of inertia of the atoms in the protein from their center of mass during a particular time interval (Kumari and Dalal, 2021). The Rg plot (Supplementary Figure S4) showed that, initially, the Rg of all the complexes and native protein was in the range of 2–2.2 nm. After 70–75 ns, the Rg of native protein decreased to an average of 1.66 nm, while complexes were found to be higher at approximately 2 nm. This shows that the ligand binding has decreased the compactness of the protein.

##### 3.2.7.4 Solvent Accessible Surface Area

The polar and non-polar interactions of the protein determine its surface area, which is known as the Solvent Accessible Surface Area (SASA). SASA of a protein decreases when there is an increase in compactness of a protein (Kumari and Dalal, 2021). The SASA plot (Supplementary Figure S5) showed that, after 100 ns, the complexes had slightly higher values than the native protein. The complex with ZINC000103666966 was found to have slightly less SASA than the other complexes. This clearly affirms that the complexes are less compact than the native protein.

##### 3.2.7.5 H-bonds

In order to determine the stability of the complexes, hydrogen bond numbers and distribution were examined during the molecular simulation of 200 ns. It was observed that the number of H-bonds was very low in the complexes, showing that the binding is based on interactions other than H-bonds like hydrophobic, van der Waals, electrostatic interactions etc.

##### 3.2.7.6 Energy

Different energies during complete dynamics were obtained, and it was found that potential energy remained negative, kinetic energy remained positive, and total energy was observed to be negative throughout the dynamics. Out of the three, the complexes with molecules ZINC000103666966 and ZINC000020413317 were found to have less potential energy, more kinetic energy, and less overall total energy compared to the complex with molecule ZINC000030883119. In comparison with the complex with molecule ZINC000020413317, the complex with molecule ZINC000103666966 was found to have less potential energy, more kinetic energy, and less overall total energy. This indicates the stability of the complex with molecule ZINC000103666966 during MD simulations.

##### 3.2.7.7 MMPBSA

The interaction energy indicates the strength of a protein–ligand complex. This was validated by estimating the binding free energy using the MM-PBSA approach (Singh et al., 2022). The MD trajectories of the 10-ns range were used to predict the binding free energy of complexes. It should be noted that the lower the binding energy, the better the interaction between the ligand and protein. In our analysis, the complex with molecule ZINC000103666966 was found to have least binding energy (−55.4 ± 5.46 kcal/mol) followed by ZINC000020413317 (−45.29 ± 7.88 Kcal/mol) and ZINC000030883119 (−34.44 ± 4.52 Kcal/mol) as shown in Table 5. This indicates that the molecule ZINC000103666966 was tightly bound to the receptor.

**TABLE 5:** Table showing the van der Waal, electrostatic + polar solvation, SASA, and binding energy in Kcal/mol of the complexes generated by MMPBSA.

Complex	∆ VDW WAALS	∆ EEL + ∆ EPB	∆ ENPOLAR	∆ G
Domain3-ZINC000103666966	−34.38 ± 2.81	2.54 ± 0.90	−26.52 ± 3.17	−55.4 ± 5.46
Domain3-ZINC000020413317	−35.9+/3.56	2.91 ± 1.77	−27.92 ± 2.20	−45.29 ± 7.88
Domain3-ZINC000030883119	−20.27 ± 2.14	0.88 ± 1.44	−19.16 ± 1.72	−34.44 ± 4.52

∆ VDW WAALS = ∆ Van der Waals energy; ∆ EEL + ∆ EPB = ∆ Electrostatic energy + ∆ Polar Energy; ∆ ENPOLAR = ∆ SASA energy; ∆ G = ∆ Binding Energy

## 4 Discussion

As there are reports suggesting the occurrence of SARS-CoV-2 interaction in different animals along with humans, our study is mainly focused on the identification of the susceptibility of SARS-CoV-2 infection in animals and the identification of a potential inhibitory natural compound against the virus. Here, we modeled the ACE2 receptors of sixteen different organisms belonging to 12 orders of mammalian (Artiodactyla, Perrisodactyla, Chiroptera, Rodentia, Carnivora, Primates, Pholidota, and Proboscidea), reptilian (Testudines and Crocodilia), and avian (Acciptriformes and Galliformes) classes. The 16 modeled 3D structures of ACE2 were validated *in silico* by the SAVES web server. The results showed that the modeled structures qualified in all the parameters. Then, we docked these structures separately to spike of SARS-CoV-2. MD simulations were performed on these complexes, and RMSD, potential energy, kinetic energy, and total energy were predicted. Based on the RMSD plot, *Chelonia mydas* was found to have a more stable ACE2–spike complex than other organisms in the study, followed by *Capra hircus* and *Manis javanica*. In other organisms, after 25 ns, *Gallus gallus* and *Rhinolophus ferrumequinum* were found to show stable RMSD between 0.9 and 1 nm. After 30 ns, *Ovis aries* showed RMSD between 0.6 and 0.8 nm and *Bos taurus* showed RMSD between 1 and 1.1 nm, while *Bubalus bubalis*, *Aquila chrysaetos chrysaetos*, *Crocodylus porosus*, and *Loxodonta africana* were found to be the least stable complexes with more fluctuations than all other organisms. Along with that, potential and total energies were found to be negative, and kinetic energy was found to be positive for all the organisms.

Apart from that, we predicted 14 domains in the orf1ab of SARS-CoV-2 and performed a sequence analysis study to identify the similarities with 16 different host organisms. On the basis of the similarities with the host proteome and annotation of the domains, Domain 3 ((DNA/RNA polymerases domain) was selected and modeled for further analysis. The modeled 3D structure of domain 3 was validated *in silico* by geometrical conformations and stereochemical quality assessments. The Ramachandran plot of the modeled structure of domain 3 indicated that all the residues were in the allowed region. Validation results from SAVES showed that the modeled structure has qualified in all the parameters and thus, Domain 3 was proceeded further for Docking.

The domain 3 was then docked with ∼1.8 lakh natural compounds from the ZINC database, and the top 10 molecules, based on binding energy, were selected. Along with this, ADME and toxicity analysis were performed on the top 10 natural compounds. Based on the ADME results, three molecules, ZINC000020413317, ZINC000103666966, and ZINC000030883119, were selected. Also, these three selected molecules were non-toxic. Structural analysis was performed, and the complex with molecules ZINC000020413317, ZINC000103666966, and ZINC000030883119 was predicted to be harbored inside the pocket. MD simulations explored the stability of the three selected molecules with domain 3 of orf1ab of SARS-CoV-2. In post-simulation analysis, the RMSD plot showed that all the three complexes attained stability after 50 ns till 200 ns. Of them, the complex with molecule ZINC000103666966 was found to have fewer fluctuations in RMSD than the complexes with the other two molecules and was close to the RMSD of the native protein. This shows that the molecule binds properly to Domain 3 with fewer RMSD fluctuations, which specifies the overall integrity of the complex. The RMSF plot showed that most of the residues in native protein had higher fluctuations than those in the complexes, showing that binding of ligands had decreased the fluctuations in the protein, were well-fitted, and formed a stable complex. Apart from that, the residues involved in the binding were also found to be less fluctuant in the complex than in the native protein. The Rg analysis showed that the ligand binding has decreased the compactness of the protein and the SASA analysis clearly affirms that the complexes are less compact than the native protein. The complex with ZINC000103666966 was found to be having slightly lower Rg and SASA values than the other complexes, which shows that this complex is more stable than the other complexes. In addition, the complex with molecule ZINC000103666966 was also found to be having less potential energy, more kinetic energy, and less overall total energy in comparison to others two complexes. The binding free energy calculations (MMPBSA) also affirm the stability of the complex of Domain 3 with the ZINC000103666966 molecule as it showed the least binding energy of all the three complexes. This shows the stability of the complex of Domain 3 and the ZINC000103666966 molecule. Thus, the molecule ZINC000103666966 was predicted to be the most suitable inhibitory compound against Domain 3 (DNA/RNA polymerases domain) of SARS-CoV-2. We hypothesize that inhibiting Domain 3 could impact the replication of the virus, leading to a control of the viral load. This *in silico* work may be useful for creating potential treatments for SARS-CoV-2 infection, which requires additional *in vitro* and *in vivo* research before using anti-COVID-19 medications.

## 5 Conclusion

Our study is mainly focused on SARS-CoV-2 infection in animals and humans and the identification of a potential inhibitory natural compound against the virus. This study explored ACE2 receptors—spike protein complex interactions and hidden structural insights of orf1ab. From this analysis, we observed that *Chelonia mydas* was more susceptible to SARS-CoV-2 than other host organisms in the study, including humans, while *Bubalus bubalis*, *Aquila chrysaetos chrysaetos*, *Crocodylus porosus*, and *Loxodonta africana* were found to be the least stable complexes with more fluctuations than all other organisms. Apart from that, we observed that the molecule “ZINC000103666966” has a stable complex with domain 3 of orf1ab of SARS-CoV-2. The domain 3 (RNA/DNA polymerase) plays a vital role in viral replication. Thus, we hypothesize that this molecule potentially inhibits RNA/DNA polymerase, thus inhibiting viral replication. This clearly opened a new dimension in selecting a drug target from orf1ab for SARS-CoV-2. The outcomes additionally demonstrated the potential of bioinformatic technology for the analysis and forecasting of SARS-CoV-2 therapeutic candidates. Future *in vitro* and *in vivo* experimental studies need to be carried out to unravel the potential and confirm the efficacy of the proposed natural molecule against SARS-CoV-2. This study could be helpful for future researchers to work with precise target molecules.

## Data Availability

The original contributions presented in the study are included in the article/Supplementary Material; further inquiries can be directed to the corresponding author.

## Supplementary material

SUPPLEMENTARY FIGURE S1 Figure showing the similarity of the proteome of 16 organisms (comprising 12 orders) against 14 domains of orf1ab with the domain number on the X-axis and the number of hits on the Y-axis.

SUPPLEMENTARY FIGURE S2 Figure showing the pocket of the receptor (Domain 3) harboring the three molecules. Protein is in yellow color, pocket is in blue color, ZINC000103666966 is in red color, ZINC000030883119 is in green color, and ZINC000020413317 is in white color.

SUPPLEMENTARY FIGURE S3 Figure showing the graph of RMSF of complexes of Domain 3 with molecules (molecules ZINC000103666966, ZINC000020413317, and ZINC000030883119) at 200 ns time point.

SUPPLEMENTARY FIGURE S4 Figure showing the graphs of Radius of gyration of complexes of Domain 3 with molecules (molecules ZINC000103666966, ZINC000020413317, and ZINC000030883119) at 200 ns time point.

SUPPLEMENTARY FIGURE S5 Figure showing the graph of SASA of complexes of Domain 3 with molecules (molecules ZINC000103666966, ZINC000020413317, and ZINC000030883119) at 200 ns time point.

## Abbreviations

SARS-CoV-2, Severe Acute Respiratory Syndrome-Coronavirus-2; ACE2, Angiotensin Converting Enzyme 2; MERS, Middle East Respiratory Syndrome; NCs, natural compounds; GRAMM-X, Global RAnge Molecular Matching–X; ADME, Adsorption, Distribution, Metabolism, and Excretion; GROMACS, GROningen MAchine for Chemical Simulations; CHARMM, Chemistry at Harvard Macromolecular Mechanics; MDS, Molecular Dynamics Simulation; RMSD, Root Mean Square Deviation; AMBER, Assisted Model Building with Energy Refinementa; MMPBSA, Molecular Mechanics Poisson–Boltzmann Surface Area.
