# Supplementary figures and images for: In silico prediction of the animal susceptibility and virtual screening of natural compounds against SARS-CoV-2: Molecular dynamics simulation based analysis
Source: Front Genet. 2022 Aug 30;13:906955. doi: 10.3389/fgene.2022.906955 (PMC9468858; doi:10.3389/fgene.2022.906955)

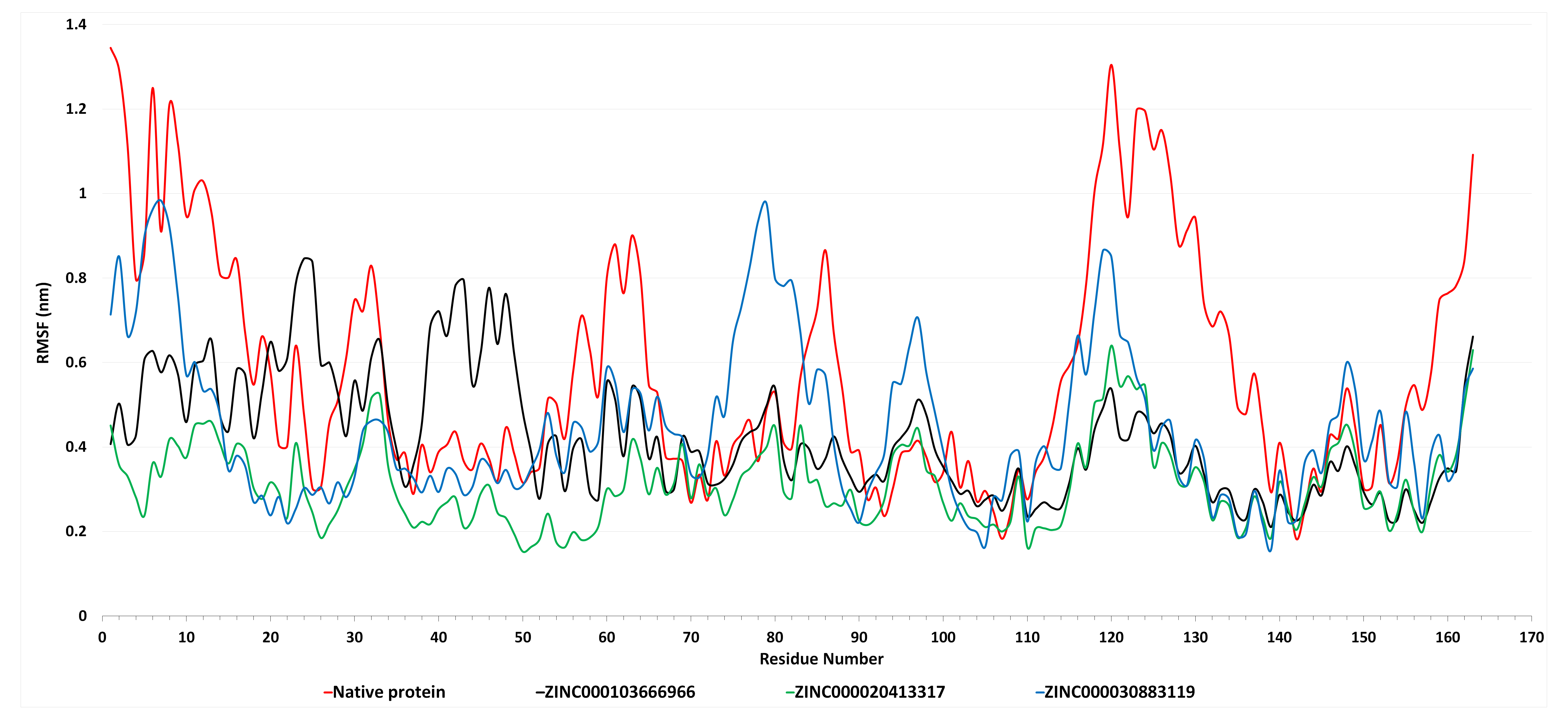

Supplement: Supplementary file 3 [file Image3.TIF]

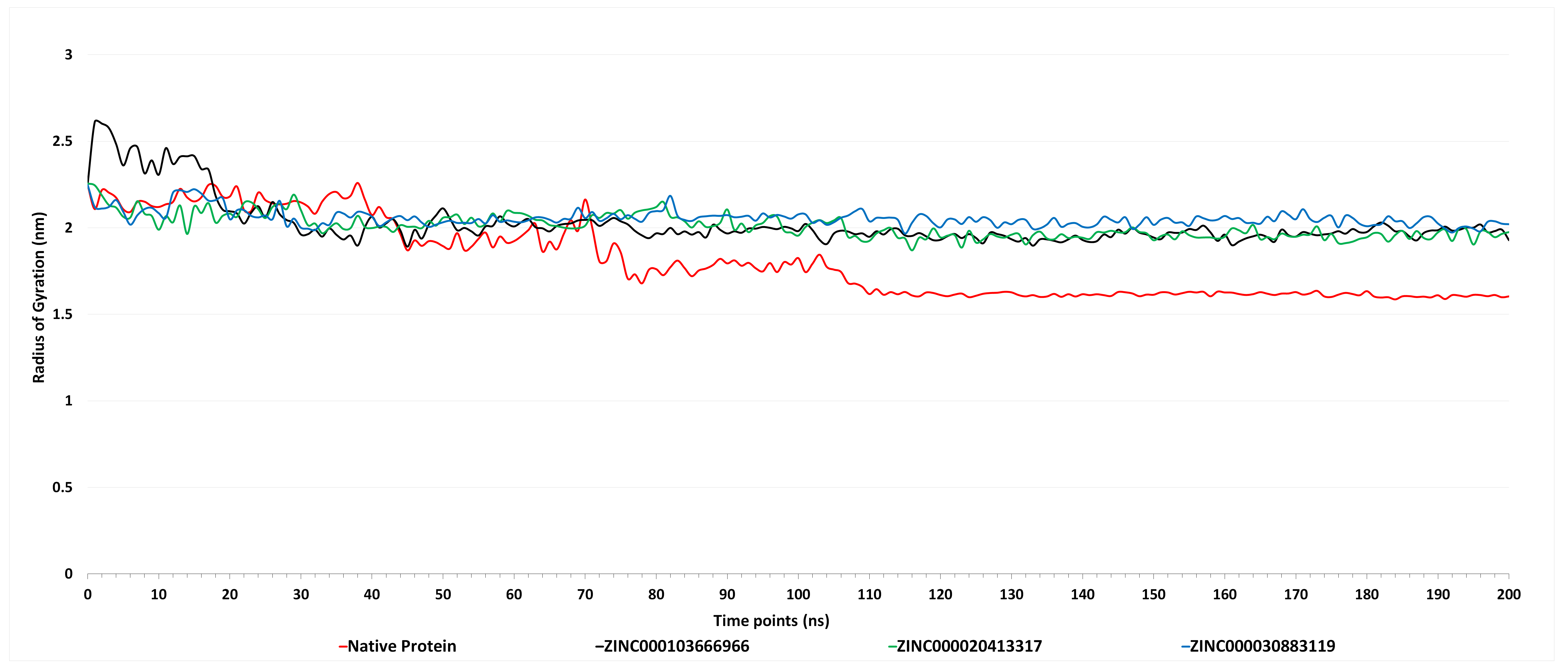

Supplement: Supplementary file 4 [file Image4.TIF]

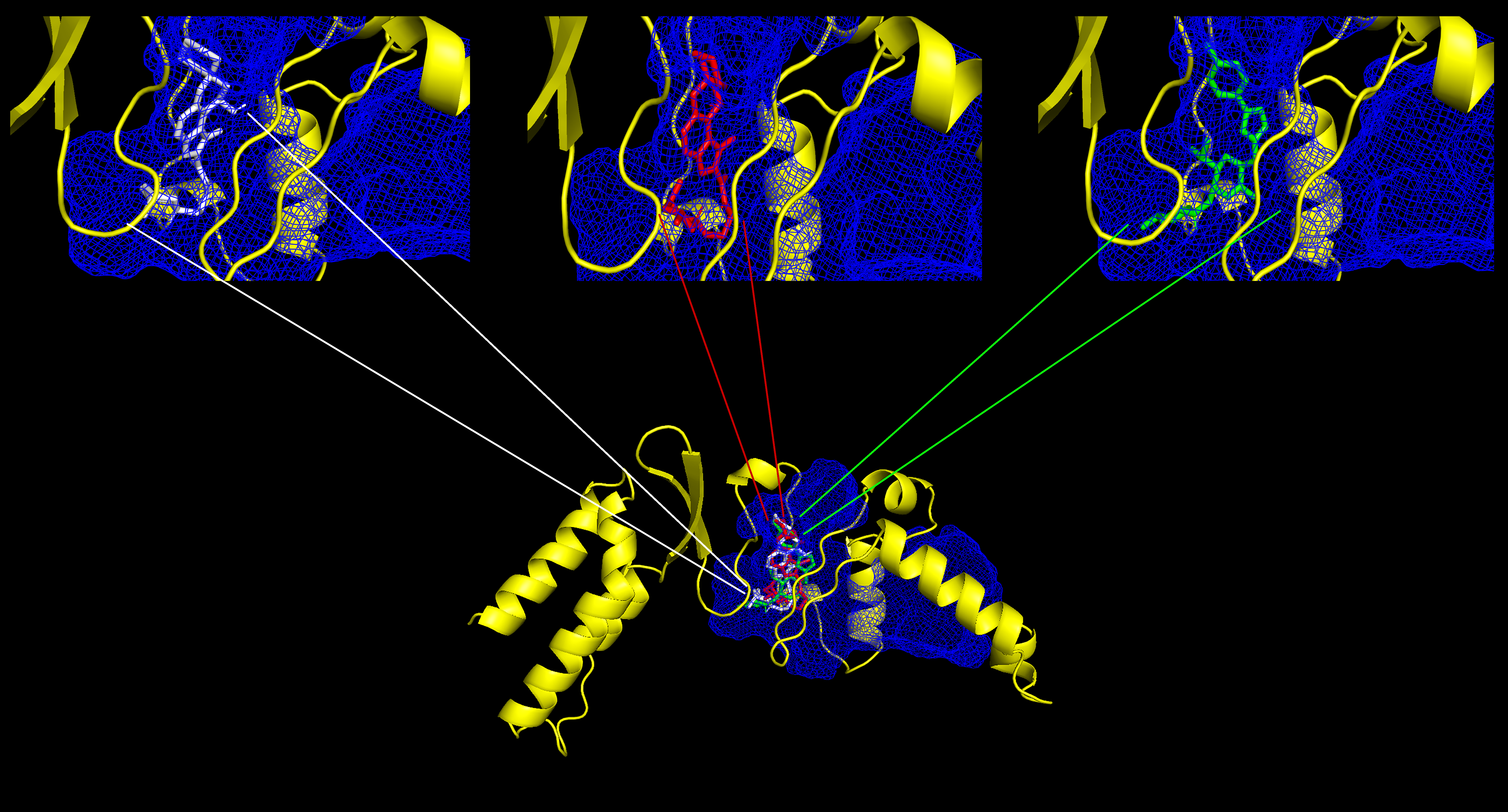

Supplement: Supplementary file 5 [file Image2.TIF]

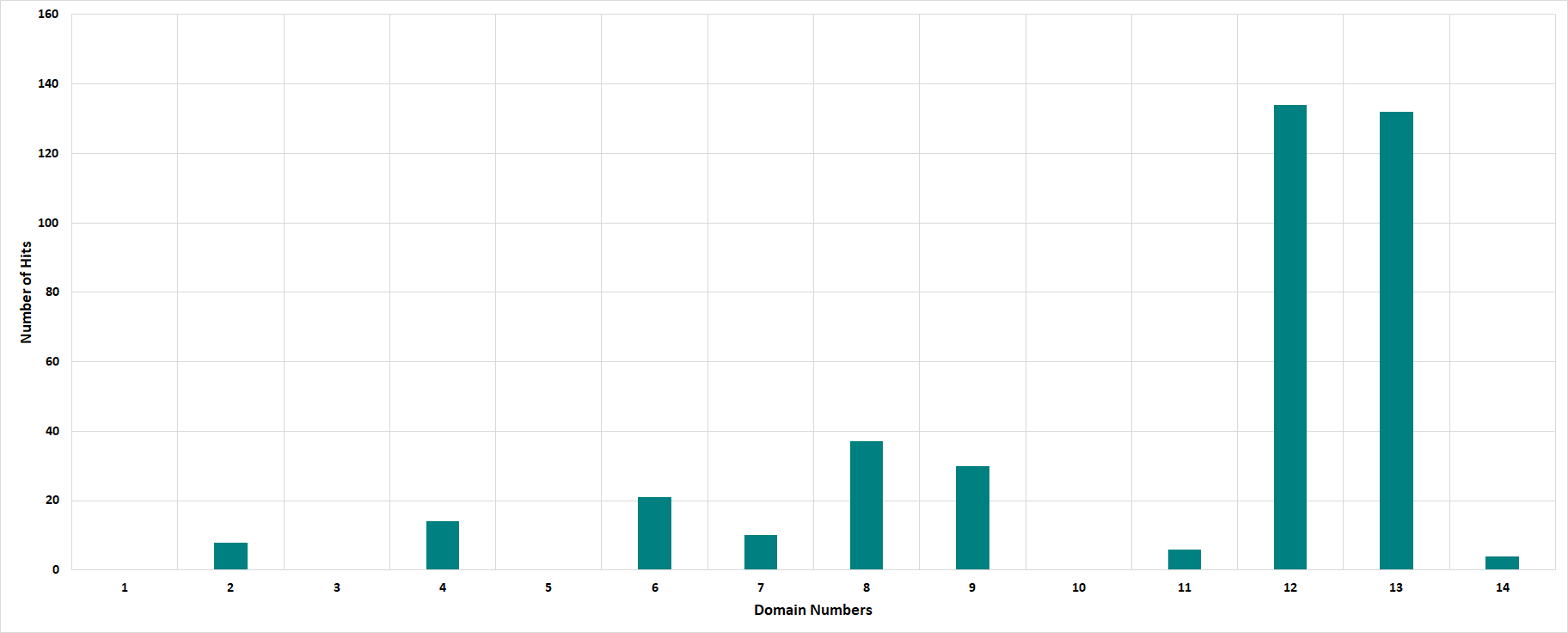

Supplement: Supplementary file 6 [file Image1.TIF]

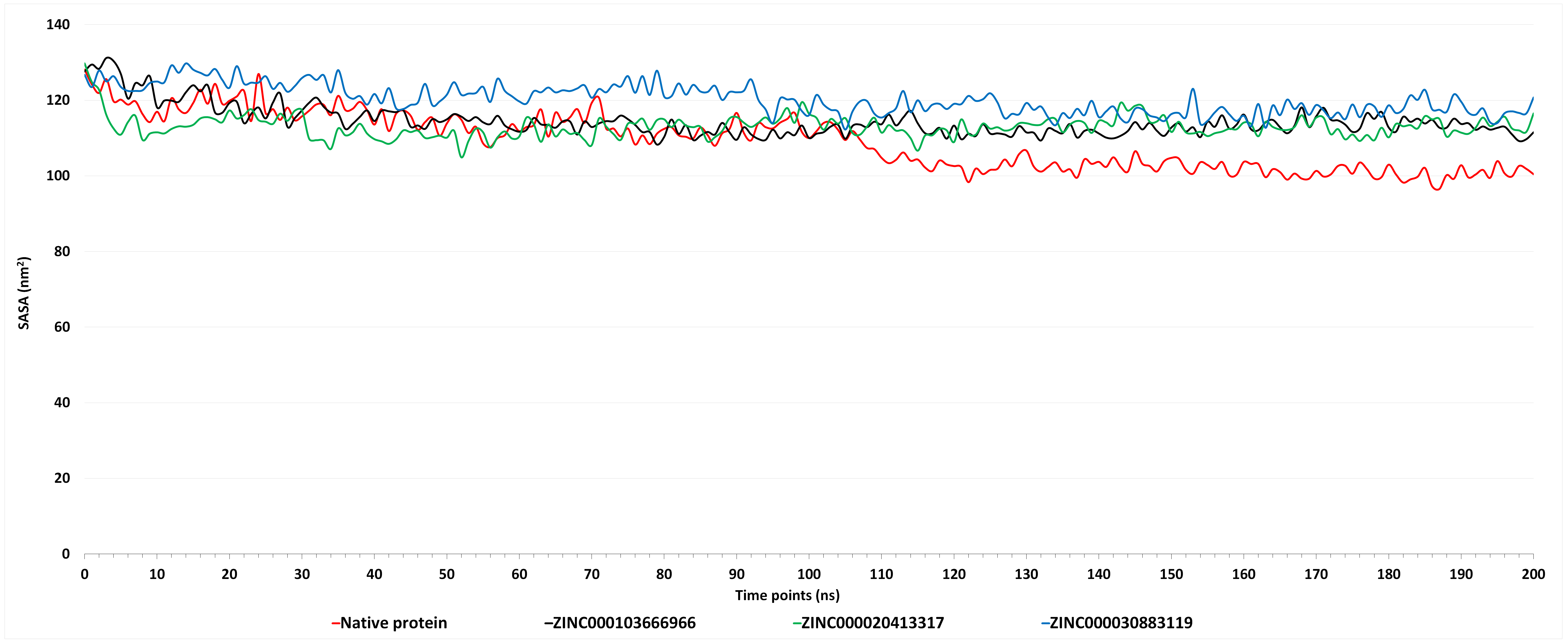

Supplement: Supplementary file 9 [file Image5.TIF]
